# A 3D Cell Death Assay to Quantitatively Determine Ferroptosis in Spheroids

**DOI:** 10.3390/cells9030703

**Published:** 2020-03-13

**Authors:** Robin Demuynck, Iuliia Efimova, Abraham Lin, Heidi Declercq, Dmitri V. Krysko

**Affiliations:** 1 Cell Death Investigation and Therapy Laboratory, Department of Human Structure and Repair, Ghent University, 9000 Ghent, Belgium; Robin.Demuynck@UGent.be (R.D.); Iuliia.Efimova@UGent.be (I.E.); 2 Tissue Engineering and Biomaterials Group, Department of Human Structure and Repair, Ghent University, 9000 Ghent, Belgium; heidi.declercq1@kuleuven.be; 3 Cancer Research Institute Ghent, 9000 Ghent, Belgium; 4 Plasma, Laser Ablation and Surface Modelling Group, University of Antwerp, 2610 Wilrijk, Belgium; abraham.lin@uantwerpen.be; 5 Center for Oncological Research, University of Antwerp, 2610 Wilrijk, Belgium; 6 Tissue Engineering lab, Department of Development and Regeneration, KU Leuven, 8500 Kortrijk, Belgium; 7 Department of Pathophysiology, Sechenov First Moscow State Medical University, 119146 Moscow, Russia

**Keywords:** ferroptosis, spheroids, 3D cultures, cell death assay, cancer

## Abstract

The failure of drug efficacy in clinical trials remains a big issue in cancer research. This is largely due to the limitations of two-dimensional (2D) cell cultures, the most used tool in drug screening. Nowadays, three-dimensional (3D) cultures, including spheroids, are acknowledged to be a better model of the in vivo environment, but detailed cell death assays for 3D cultures (including those for ferroptosis) are scarce. In this work, we show that a new cell death analysis method, named 3D Cell Death Assay (3DELTA), can efficiently determine different cell death types including ferroptosis and quantitatively assess cell death in tumour spheroids. Our method uses Sytox dyes as a cell death marker and Triton X-100, which efficiently permeabilizes all cells in spheroids, was used to establish 100% cell death. After optimization of Sytox concentration, Triton X-100 concentration and timing, we showed that the 3DELTA method was able to detect signals from all cells without the need to disaggregate spheroids. Moreover, in this work we demonstrated that 2D experiments cannot be extrapolated to 3D cultures as 3D cultures are less sensitive to cell death induction. In conclusion, 3DELTA is a more cost-effective way to identify and measure cell death type in 3D cultures, including spheroids.

## 1. Introduction

The failure rates of new therapies during translation from preclinical research to clinical trials remain a big issue in cancer research [1]. This is largely due to limitations of the two-dimensional (2D) cell cultures, the most commonly used models in drug screening. The 2D cultures do not properly resemble the structures found in vivo as they often lack cell extracellular matrix (ECM) and cell-cell interactions, thereby influencing cell death responses to therapy (in particular to apoptosis) [2,3]. In recent years, the focus of drug screenings has shifted towards the use of three-dimensional (3D) culture methods [4,5,6] as they more closely mimic the in vivo tumour microenvironment due to cell secretion of its own ECM and the formation of cell-cell interactions [7]. Therefore, 3D structures better resemble the structure and phenotype of the in vivo organ they are derived from [8,9,10,11]. Moreover, 3D structures, such as spheroids, better represent in vivo drug resistance, as they form a more dense barrier for the drugs to cross, compared to 2D systems [12], with the core of the structure being most protected against the drugs. Therefore, with regard to the responses to therapy, including to cell death, 3D structures are better test models for drug screening compared to 2D monolayers [13].

**Figure 1:**
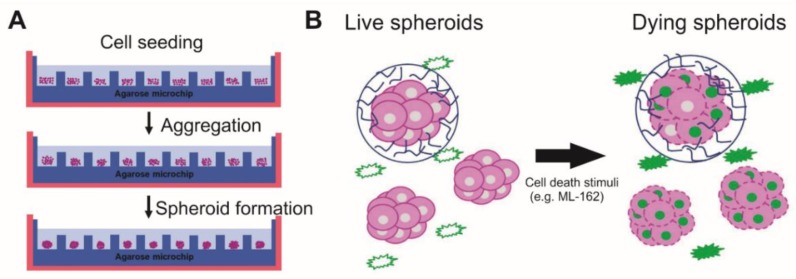
Principles of three-dimensional cell death assay (3DELTA). (**A**) Spheroids are formed by seeding cells on top of an agarose microwell chip. This chip is made by pouring liquid agarose on top of a polydimethylsiloxane (PDMS) mould. Cells will form clusters, leading to the formation of spheroids. (**B**) Sytox Green is nonfluorescent outside of viable cells. When cells are permeabilized at the end of the cell death process and the plasma membrane ruptures, Sytox Green will bind to the DNA and emit green fluorescence, which can then be measured.

Spheroids are one of the most widely used 3D models and can be formed by seeding cells in nonadherent plates allowing for self-assembly (Figure 1A) [7]. In spheroids, the ECM can be produced by cells, which acts as an extra barrier for drugs to cross in order to induce tumour cell death [14,15,16]. Moreover, the compactness and stiffness of spheroids resemble the in vivo hypoxic tumour microenvironment [17], which can cause drug resistance through cell cycle arrest and downregulation of pro-cell death proteins (e.g., caspase-3 in apoptosis) [18]. Altogether, spheroids can be more easily formed in a high-throughput manner compared to other 3D models and better resemble in vivo conditions, thus making them an attractive model for more efficient drug screening and analysis of cell death responses.

Of interest, in the past, cancer cell death was perceived to be dichotomic with only two ways for a cell to die—in a regulated way (apoptosis, controlled via caspase-activation and cytochrome c release [19]) or in an uncontrolled way (accidental necrosis) [20,21]. In line with this concept, necrosis was seen as a form of unprogrammed cell death from cellular damage, which could be induced by different acute physical-chemical injuries (e.g., heat shock, detergents). However, in the past few decades, research has indicated more types of regulated cell death besides apoptosis, which are assembled under the umbrella term ‘regulated necrosis’ [22,23]. One example of regulated necrosis is called ferroptosis, a newly discovered pathway in cell death, which may be a potential alternative therapeutic pathway to overcome apoptosis-resistant cancer. Ferroptosis can be induced through blockage of Glutathione Peroxidase 4 (GPX4), an important inhibitor of ferroptotic cell death [24,25,26].

Of note, cell death has been thoroughly studied in 2D cultures, and despite the acceptance that 3D cultures including spheroids are better models for the in vivo environment, not many cell death assays exist for 3D cultures, even less for ferroptosis as the translation of the assay between culture systems is not straightforward. Most commonly used cell death assays in 2D include Calcein AM-propidium iodide (PI) stainings [27], immunohistochemical staining (for example Caspase-3 staining for apoptotic cells [7]), LDH- and ATP-based assays or flow cytometry for the detection of membrane and intracellular markers of cell death [28,29,30]. However, each of these assays present their own disadvantages for high-throughput cell death response screening in 3D cultures. For example, in Calcein AM-PI staining, PI ions can enter viable cells with a high membrane potential which in turn leads to false positive results [31]. For live/dead assays, such as Calcein AM-PI, the interior of the spheroids cannot be seen with ordinary fluorescence microscopy and require sectioning [28], a labour-extensive process. For immunohistochemical staining, sectioning is also required, making this method cumbersome when monitoring over time is needed [32]. ATP may be used as an indicator for cell viability, but the signal received from ATP luminescence assays is not stable and therefore not reliable [33]. Moreover, as a preparation step, the cells need to be lysed to extract the ATP and measure luminescence, thereby restricting the assay to endpoint analysis. Flow cytometry is another endpoint assay that can only be performed on single cells. Thus, spheroids must be disaggregated, a process and technique requiring extensive training before proficient use [34].

New tools are being developed to overcome the current challenges with 3D analysis, but a simple, quantitative method of determining real-time cell death modalities in 3D cultures remains difficult [35]. Therefore, the aim of this study is to develop a reliable assay which will allow for quantitative determination of cell death type in spheroids. In this study, spheroids were formed by seeding tumour cells (L929 mouse fibrosarcoma and SK-OV-3 human ovarian cancer cells) on top of nonadherent agarose microwell chips and allowing them to self-assemble. The cell death assay was optimized using spheroids collected on day 1. To validate the identification of cell death type, ferroptosis was induced in spheroids collected on day 1 and 10, in the presence or absence of different cell death inhibitors (zVAD-fmk for apoptosis, necrostatin-1s (Nec-1) for necroptosis and ferrostatin-1 (Fer-1), deferoxamin (DFO) and α-tocopherol (α-Toc) for ferroptosis). In this work we demonstrated that this method, named the 3D Cell Death Assay (3DELTA), can efficiently and quantitatively determine different cell death types, including ferroptosis in tumour spheroids. 3DELTA will make it possible to investigate high-throughput cell death responses to therapy with tumour spheroids. The results from this assay and 3D model will be more representative of therapy response in the clinic, and improve the rate of successful drug translation into clinical trials.

## 2. Material and Methods

### 2.1. Cell Lines and Culture

L929sAhFas mouse fibrosarcoma cells were cultured in Dulbecco’s Modified Eagle Medium (DMEM) Glutamax (Life Technologies, Carlsbad, CA, USA), 10% foetal calf serum (FCS, Life Technologies) and 1% Penicillin/Streptomycin (Life Technologies) at 37 °C and 5% CO_2_.

SK-OV-3-luc-GFP human ovarian cancer cells (kindly provided by LECR-UGent) were cultured in DMEM Glutamax containing 10% FCS, 1% Penicillin/Streptomycin at 37 °C and 5% CO_2_. In this study for convenience, the L929sAhFas and SK-OV-3-luc GFP cell lines have been abbreviated to L929 and SKOV, respectively.

### 2.2. Spheroid Formation

An Ultrapure Agarose solution (3% *w*/*v*, Life Technologies) was dissolved in sterile PBS and heated. After heating, it was poured on top of a negative polydimethylsiloxane (PDMS) mould which has a diameter of 18 mm and a height of 3 mm (NaMiFab, Ghent University, 9000 Ghent, Belgium). Each microwell contains 2865 pores with a diameter of 200 µm and a depth of 220 µm each. At room temperature, the solution solidified and the agarose microwell was separated from the mould. The microwell was then placed in a 12-well culture plate. A total of 500 µL of the cell suspension, containing 1 × 10^6^ cells, was seeded onto the microwell, which resulted in approximately 349 cells per pore. Either L929 or SKOV cells were used for spheroid formation and spheroids were cultured at 37 °C in a humidified 5% CO_2_-containing incubator. Medium was refreshed after 24 h of culture and afterwards every two days. Either day 1 and 10 spheroids (L929) or only day 1 spheroids (SKOV) were used.

### 2.3. Sytox Dyes

Sytox dyes intercalate with DNA with a high affinity [36]. When cells are permeabilised (i.e., the plasma membrane is ruptured) at the end of any cell death process, Sytox enters the cell and binds to the DNA causing a large increase in fluorescence (quantum yield of 0.5, Figure 1B) which can be measured via a fluorescence microplate reader and the Sytox signal can be correlated to the percentage of cell death. Both Sytox Green and Sytox Blue were used for L929 and SKOVs, respectively.

### 2.4. Fluorescence Microscopy and Morphological Assessment of Spheroids

Induced spheroids were evaluated using an inverted fluorescence microscope (Olympus IX81). For L929 spheroids, a GFP filter was used to visualize Sytox Green (λ_ex_ = 485 nm, λ_em_ = 520 nm) while for SKOV-spheroids, a DAPI filter was used to visualize Sytox Blue (λ_ex_ = 444 nm, λ_em_ = 480 nm).

The geometry of spheroids was analysed using Xcellence image software (Olympus). Diameter (d), area (A) and perimeter (p) were measured after 1, 2, 5, 7 and 10 days of culture and sphericity was calculated using Excellence image software. Volume was calculated using the formula $V=\left(\pi d^{3}\right)/6$. Images of 23–29 spheroids were analysed for diameter, circularity and volume.

### 2.5. Identification of Optimal Sytox Concentration

L929- and SKOV-spheroids were collected at day 1 and seeded in different densities (ranging from 3 to 120 spheroids per well) in a 96-well plate in duplicates. A 2D control was seeded as well in different densities (ranging from 500 to 20,000 cells per well). Cells were stained either immediately after seeding (spheroids) or after 24 h (2D culture) using different concentrations of Sytox Green (ranging from 1 to 5 µM) for L929-spheroids and Sytox Blue (ranging from 2.5 to 5 µM) for SKOV spheroids and after 2 h spheroids were permeabilised with 10 µL Triton X-100 (0.05% *v*/*v*, MERCK, Darmstadt, Germany). After 2 h, Sytox intensity was measured using the Tecan Spark^®^ (Tecan, Männedorf, Switzerland) 20M microplate fluorescence reader (Sytox Green: λ_ex_ = 485 nm, λ_em_ = 535 nm; Sytox Blue: λ_ex_ = 430 nm, λ_em_ = 460 nm).

### 2.6. Determining the Optimal Timing of Triton X-100 Treatment and Triton X-100 Concentration

L929-spheroids were collected at day 1 and seeded in different densities (30, 80 and 120 spheroids per well) in a 96-well plate in triplicate. A 2D control was seeded as well in different densities corresponding to the densities of cells present in the spheroids (10,620, 28,000 and 48,000 cells per well). Cells were stained either immediately after seeding (spheroids) or after 24 h (2D culture) with the optimal Sytox Green concentration (found in the previous experiment, Invitrogen, Carlsbad, CA, USA) and permeabilised with different concentrations of Triton X-100 (0.05% *v*/*v*, 0.10% *v/v* and 0.25% *v*/*v*) in a volume of 10 µL added to the medium. At different timepoints (each hour, starting from 2 h), Sytox Green intensity was measured using the Tecan Spark microplate fluorescence reader.

### 2.7. Validation of 3DELTA: Quantification of Ferroptotic Cell Death

Spheroids were collected at day 1 and 10 (L929) and day 1 (SKOV) and seeded at a density of 80 spheroids per well in a 96-well plate in duplicate. A 2D control was seeded as well at a density of 10,000 cells per well. Cells were stained either immediately after seeding (spheroids) or after 24 h (2D culture) using the optimal Sytox Green (L929) or Sytox Blue (SKOV) concentration. An apoptosis inhibitor (zVAD-fmk, BACHEM, Budendorf, Switzerland), necroptosis inhibitor (Nec-1s, Abcam, Cambridge, UK) and multiple ferroptosis inhibitors (Fer-1, DFO and α-Toc, Sigma, Saint Louis, MO, USA) were added 30 min before cell death induction with 5 µM ML-162 (AOBIOUS, Gloucester, MA, USA), an inhibitor of GPX4 and thus an inducer of ferroptosis. After 24 h, Sytox intensity was measured using the Tecan Spark microplate fluorescence reader. Afterwards, cells were permeabilised with the optimal Triton X-100 concentration (found in the previous experiment) to obtain 100% of cell death. After 2 h of incubation, Sytox intensity was measured again.

### 2.8. Visualization of Cell Death Pattern

Sytox fluorescence intensities were measured using the Tecan Spark^®^ 20M microplate multimode reader. Each well of the 96-well plate was scanned in a ‘5 × 5 filled pattern’ for a total of 21 positions per well. A script was written in Matlab (R2018b, Mathworks, Natick, MA, USA) to extract the fluorescence intensity data from the Tecan output file and a heat map was constructed using the pseudocolour plot function. Shading of each pixel of the image was based on the measured fluorescent intensity at specific positions in the well. Corners of the image have zero values as these areas were not included in the Tecan Spark-defined scan pattern.

### 2.9. Statistical Analysis

The percentage of the cell death is calculated by the following formula:

(1) $$\frac{average_{Sytox}\left[ML162\right]-average_{Sytox}\left[background\right]}{average{\;}_{Sytox}\left[Triton\;X-100\right]-average_{Sytox}\left[background\right]}*100\%$$

Cell death data are analysed as repeated measurements. Data from 3 independent experiments were analysed. Statistical analysis is performed using GraphPad Prism 8.0 software and are represented by mean ± SEM. Data are analysed with two-way ANOVA. *p*-values less than 0.05 are considered significant.

## 3. Results

### 3.1. Linear Correlation between Sytox Intensity and Cell Density

**Figure 2:**
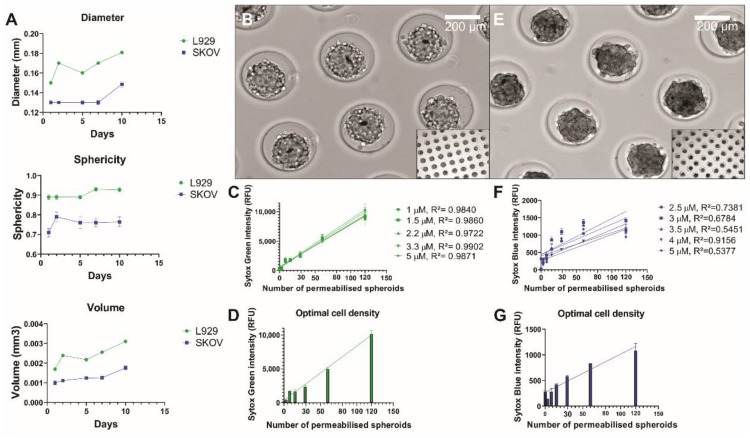
Characterization of the spheroids and analysis of linear correlation between Sytox intensity and cell density. (**A**) Diameter, sphericity and volume of L929 and SKOV spheroids. (**B**) Brightfield image of day 1 L929 spheroids showing globular spheroids that are not yet fully compacted. (**C**) Sytox Green intensity for different concentrations of Sytox Green was plotted out against different spheroid densities. Linear correlation was measured as R^2^ and the highest correlation was found for Sytox Green 3.3 µM. (**D**) The increase of Sytox intensity for Sytox Green 3.3 µM is shown. Starting from 30 spheroids, a high increase in intensity was found until 120 spheroids. (**E**) Brightfield image of day 1 SKOV spheroids showing irregularly shaped spheroids that are quite compact. (**F**) Sytox Blue intensity for different concentrations of Sytox Blue was plotted out against different spheroid densities. Linear correlation was measured as R^2^ and the highest correlation was found for Sytox Blue 4 µM. (**G**) The increase of Sytox intensity for Sytox Blue 4 µM is shown. Starting from 30 spheroids, a high increase in intensity was found until 120 spheroids.

First, the L929 and SKOV spheroid geometry was characterized over time (Figure 2A). The diameter, sphericity and volume were measured and calculated for day 1, 2, 5, 7 and 10. L929 diameter increased from an average diameter of 150 ± 1.30 µm on day 1 to 180.83 ± 1.03 µm on day 10 and SKOV diameter increased from 130 ± 1.63 µm on day 1 to 148.44 ± 2.81 µm on day 10. Sphericity remained stable around 90% and 75% for L929 and SKOV spheroids, respectively. The volume increase over time of spheroids correlates with an initial compaction phase followed by the proliferative phase of cancerous cells [37].

In order to correctly extrapolate the percentage of cell death over time, a linear correlation between the cell number and Sytox intensity is required. Therefore, the optimal Sytox concentration was established by testing a range of concentrations from 1 to 5 µM for Sytox Green and from 2.5 to 5 µM for Sytox Blue. Of note, Sytox Blue has a lower signal-to-noise ratio than Sytox Green [38]. For 2D cultures, the optimal concentration were already determined as 3.3 µM for Sytox Green in a previous study [39] and 5 µM for Sytox Blue, which was also confirmed in this study (supplemental Figure S1A,E). For L929 spheroids (Figure 2B) the same concentration can be applied for Sytox Green, based on the linear correlation (R^2^ = 0.9902, Figure 2C). In the case of SKOV spheroids (Figure 2E), it was observed that 4 µM of Sytox Blue had the highest linear correlation with cell density (R^2^ = 0.9156, Figure 2F). These optimal concentrations were then used for all further experiments. An optimal spheroid density was determined from a range of 30 to 120 spheroids per well (Figure 2D,G). Of note, we measured a large increase of fluorescence when the number of spheroids per well was above 30. Thus, in the following experiments, spheroids were seeded at a density of 80 spheroids per well.

### 3.2. Sytox Signal Penetrates All Cells in the Spheroids

In order to obtain the maximal intensity of the Sytox dyes, which corresponds to 100% cell membrane permeabilization and cell death, Triton X-100 was used to physically rupture the membrane. Previously in 2D cultures, Triton X-100 was used at a concentration of 0.05% (*v*/*v*) in order to permeabilize the cells and establish 100% cell death (39). However, spheroids maintain a structure of higher complexity than 2D cultures (Figure 1B and Figure 2B,E). Therefore, optimization of spheroid permeabilization was performed. After staining with Sytox, spheroids were permeabilised using different concentrations of Triton X-100 (0.05%, 0.10% and 0.25%). To ensure that all cells undergo permeabilization after addition of Triton X-100, spheroids were disaggregated with trypsin. The fluorescence intensity of this 2D suspension was then compared to that of untrypsinized 3D spheroids.

**Figure 3:**
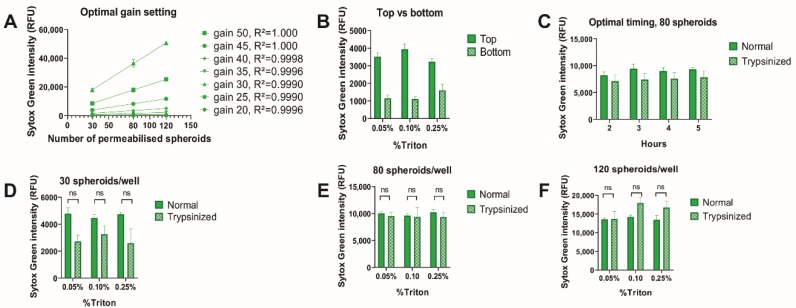
Triton X-100 efficiently permeabilizes spheroids. L929 spheroids were stained with 3.3 µM of Sytox Green and permeabilised with Triton X-100 in different concentrations (0.05%, 0.10% and 0.25%). Sytox Green intensity was measured using the Tecan Spark microplate reader. Either top or bottom measurement was used, and intensity was measured at different timepoints. (**A**) Optimal gain setting. The highest linearity is found at gain 45 and 50. (**B**) Comparison of top and bottom measurement. The most intense signal of Sytox Green with the lowest variation is found in top measurement. (**C**) Optimal timing for measurement. No differences were observed at the different timepoints. (**D**–**F**) Comparison of normal and trypsinized spheroids at different cell densities and with different Triton X-100 concentration. No significant differences in Sytox Green intensity were observed between normal and trypsinized. Furthermore, no significant differences were found between different Triton X-100 concentrations. Data are averages from three independent experiments (*n* = 3), each measured in triplicate; error bars = SEM. * *p* < 0.05, ** *p* < 0.01, *** *p* < 0.001, ns = not significant. RFU = relative fluorescent unit.

Since the optimal gain setting is an inherent characteristic of fluorescence readers, we tested this first for our machine. The optimal gain setting was established by determining the gain with the highest linear correlation between Sytox Green intensity and cell density (Figure 3A). The highest correlation was found at gain 45 and 50 (R^2^ = 1.000) and a gain 45 was used for the subsequent experiments. Next, top and bottom measurements were compared for spheroids (Figure 3B), which revealed that a more intense signal was received with less variation from top measurement, meaning that this setting was optimal for spheroid cultures. Furthermore, the incubation time with Triton X-100 required to permeabilize spheroids was tested and compared to 2D cultures (Figure 3C). Since the intensity of the Sytox signal did not change over time, this indicated that two hours was enough time to fully permeabilize spheroids with Triton X-100 at all concentrations.

The comparison between standard and trypsinized spheroids was made for 30, 80 and 120 spheroids seeded per well; using different concentrations of Triton X-100: 0.05%, 0.10% or 0.25% (Figure 3D–F). No significant differences were found between different Triton X-100 concentrations and therefore, Triton X-100 0.05% (*v*/*v*) was used to permeabilize spheroids in all subsequent experiments. Importantly, no significant differences were observed between Sytox green intensity in standard spheroids and trypsinized spheroids (*p* > 0.05, *n* = 3). This indicates both that the cell death stain can penetrate into the core of intact spheroids and that the fluorescence emission can be detected.

### 3.3. Validation of 3DELTA: Quantification of Ferroptotic Cell Death

**Figure 4:**
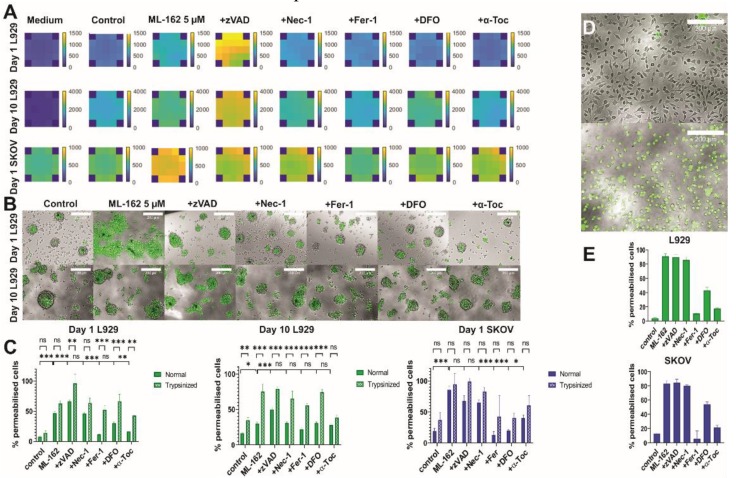
Validation of 3DELTA—quantification of ferroptotic cell death in spheroids. Ferroptosis was induced using 5 µM ML-162. Spheroids were stained with Sytox Green (L929) or Sytox Blue (SKOV) and cell death was measured after 24 hours as increase in fluorescence intensity using Tecan Spark microplate reader. Afterwards, spheroids were permeabilised with Triton X-100 0.05% (*v*/*v*). Fluorescence intensity was measured and used as 100% cell death. (**A**) Sytox was measured in a consistent, predefined pattern (21 positions per well), and heat maps of the entire well were generated in Matlab based on fluorescence intensities. Optimal gains were used for each cell line (gain 45, D1 L929; gain 45, D10 L929; gain 55, D1 SKOV). The corners shown here are zero values as these areas were not measured in the predefined Tecan Spark scan pattern. Experiments were performed in triplicate and representative images are shown here. (**B**) Fluorescence pictures of day 1 and 10 L929 spheroids. Control spheroids show cell death in their core which might be due to hypoxia leading to a necrotic core. Representative images are shown here. Scale bar = 200 µm (**C**) Quantification of cell death in normal and trypsinized spheroids. Data are averages from three independent experiments (*n* = 3), each measured in triplicate; error bars = SEM. * *p* < 0.05, ** *p* < 0.01, *** *p* < 0.001, ns = not significant. The first line represents the comparison between standard and trypsinized spheroids and the second line represents the comparison between control spheroids and spheroids induced with ML-162 and between induced spheroids and spheroids where inhibitors were added. (**D**) Brightfield image of control (upper panel) and induced (lower panel) L929 cells. All cells are stained with Sytox Green. (**E**) Quantification of cell death in L929 (upper panel) and SKOV (lower panel) 2D culture.

In order to validate cell death identification and quantification at the optimized measurement conditions for spheroids, ferroptotic cell death was induced with 5 µM ML-162, an inhibitor of GPX-4, and the 3DELTA method was performed. Different inhibitors of cell death modalities were added to confirm the type of cell death in spheroids—apoptosis (zVAD-fmk), necroptosis (Nec-1) and for ferroptosis (Fer-1, DFO and α-Toc) [40]. Visualization of cell death was performed for each well based on measured fluorescence intensities. A predefined scan pattern was used to measure Sytox intensity at specific points in the well, and a Matlab script was used to compile the data and construct heat maps. Based on the heat maps, the inhibitory effect of Fer-1, DFO and α-Toc and the distribution of cell death in each well are apparent (Figure 4A). Furthermore, it is clear that cell death induction is more efficient in day 1 spheroids compared to day 10 spheroids as well as inhibition of cell death.

Interestingly, both day 1 and 10 spheroids showed cell death in the core in control spheroids (Figure 4B) which could be due to hypoxia leading to a necrotic core [17]. ML-162 induced a significant increase of cell death to around 50% compared to control L929 spheroids that were collected at day 1 (Figure 4A,C). However, the cell death response was decreased to around 30% in day 10 spheroids. The decrease in ferroptosis could be caused by an increase in complexity of spheroids over time and secretion of extracellular matrix [16]. For day 1 SKOV spheroids, approximately 85% of cell death was observed after stimulation with ML-162. The addition of ferroptosis inhibitors (i.e., Fer-1, DFO and α-Toc) to drug-induced spheroids significantly reduced cell death, indicating that the induced cell death followed the ferroptosis pathway. However, this was less prominent with DFO, compared to Fer-1 and α-Toc. The addition of zVAD-fmk, which inhibits apoptosis, appears to increase the amount of cell death and may be due to the induction of necroptosis via inhibition of caspase-8 [41]. It has previously been shown that zVAD-fmk can induce necroptosis via induction of tumour necrosis factor-α (TNFα) through the mitogen-activated kinases (MAPKs) pathway [42]. In day 10 L929 spheroids, inhibition of ferroptotic cell death was also less pronounced, which may be due to the more complex nature at day 10, preventing efficient diffusion of inhibitors towards the centre of the spheroids.

A comparison between normal and trypsinized spheroids was also made with the inhibitors (Figure 4C). Importantly, significant differences were found between the two conditions. These differences might be explained by the stress induced by the trypsinization procedure, which leads to dysregulated cell functions [43]. This was particularly apparent in day 10 spheroids where the cells were treated with collagenase-1 for 30 min. Collagenase-1 was used to disaggregate day 10 spheroids as trypsin could not efficiently degrade the ECM produced in this more advanced stage. Already in the control group, more cell death was observed, and here, the differences between trypsinized and nontrypsinized spheroids were even more pronounced. It also means that using trypsinized spheroids might give a wrong representation of cell death induction and lead to false positive or negative results. However, in day 1 SKOV spheroids, no significant differences between standard and trypsinized spheroids was found. In contrast, there was a large variation in the percentage of permeabilised cells in trypsinized SKOV spheroids, which may also contribute to false results in this case.

## 4. Discussion

High failure rates in drug development when entering into the clinic is a large healthcare problem. A major contributing factor is due to the poor models used in early target development which are mainly in 2D models. In this work, cell death was induced in spheroids which are more representative of the in vivo environment. We show that a new cell death analysis method (i.e., 3DELTA) can efficiently determine different cell death types including ferroptosis and quantitatively assess cell death in tumour spheroids. These results obtained from 3D cultures might be more relevant to the clinic and consequently will save time, money, and resources for drug development.

This work also strongly highlights the advantage of the 3DELTA method over existing cell death measurements [44], which require disaggregation of spheroids at one specific timepoint for analysis. Not only is the disaggregation process time-consuming, it also cannot be applied for kinetic measurements as it destroys the 3D environment. With the 3DELTA method, real-time kinetic and high-throughput measurements are possible if the machine is coupled to a CO_2_ inlet and a heating control. Moreover, in this work it was shown that 2D experiments cannot be extrapolated towards 3D cultures as 3D cultures are less sensitive to cell death induction. In 2D cultures, ferroptosis induction leads to approximately 90% cell death (Figure 4E) while in day 1 spheroids this was already reduced to 50% and even to 30% in day 10 spheroids. Due to tumour heterogeneity, the responses between different tumour types might differ because of different sensitivity to cell death induction, which was also represented in this work as the SKOV cell line seemed to be more sensitive to ferroptosis [45]. These two cell lines have been chosen because they are extensively used in cell death research in 2D cultures [42,46,47]. Furthermore, use of two different cancer cell lines (murine fibrosarcoma and human ovarian cancer cell line) circumvents a lot of risks with cell type specificity of the DELTA assay. These data suggest that this method is also applicable to other cell lines. The application of 3D cultures is more representative to the in vivo situation as spheroids better resemble in vivo tumours. Moreover, ferroptosis will be an alternative strategy in cancer therapy, especially in apoptosis- and necroptosis-resistant cases [23,48]. Ferroptosis is induced via blockade of the system x_c_ cysteine/glutamate antiporter or glutathione peroxidase 4 (GPX4), resulting in a defective GSH-redox system where oxidized phosphatidylethanolamines are crucial for ferroptosis execution [26,49]. In the proposed 3DELTA method, it is possible to monitor ferroptotic cell death in time kinetics and without disaggregation of the spheroids. We were able to induce ferroptosis in 3D cancer models allowing the possibility of ferroptosis to be exploited in the future therapies. Of course, there are other important pathways which can contribute to cell death, such as autophagy. Autophagy often precedes cell death (e.g., apoptosis or ferroptosis) after which the cells will become Sytox positive [50,51]. Indeed, we have only used inhibitors for apoptosis, necroptosis and ferroptosis. However, this method can be easily adapted to include other inhibitors like autophagy inhibitors.

3DELTA proved to be a simple and efficient cell death assay to identify and quantify cell death modality in 3D cultures (i.e., spheroids) compared to the current state-of-the-art methods. It does not require the disaggregation of spheroids, making it less labour-extensive and allowing for potential real-time kinetic experiments. 3DELTA can be easily adapted to different stimuli and/or inhibitors. This is highly advantageous for high-throughput drug screenings in 96- and 384-well plates. However, several factors, summarized in Table 1, should be taken into consideration for the proper use of this assay. In summary, we have developed a more cost-effective way to measure cell death and determine its type in 3D cultures including spheroids.

## Figures and Tables

**Table 1:** Troubleshooting.

Issue	Solution
Higher Sytox intensity in stimulated cells than with Triton X-100 leading to cell death higher than 100%	Resuspend more vigorously and repeat after 1 h Increase Triton X-100 concentration
Trypsinization incomplete for D10 spheroids	Include an incubation step with collagenase I instead of using trypsin
Spheroids disaggregate after 1 day during ferroptosis assay when using adherent plates	Use suspension plate instead of adherent plates to avoid cells adhering to plate
Intensity too high: overestimated	Use lower gains Optimize gain settings for new cell lines

## Supplementary Materials

Supplementary Materials can be found at https://www.mdpi.com/2073-4409/9/3/703/s1.
