# Supplementary figures and images for: A 3D Cell Death Assay to Quantitatively Determine Ferroptosis in Spheroids
Source: Cells. 2020 Mar 13;9(3):703. doi: 10.3390/cells9030703 (PMC7140689; doi:10.3390/cells9030703)

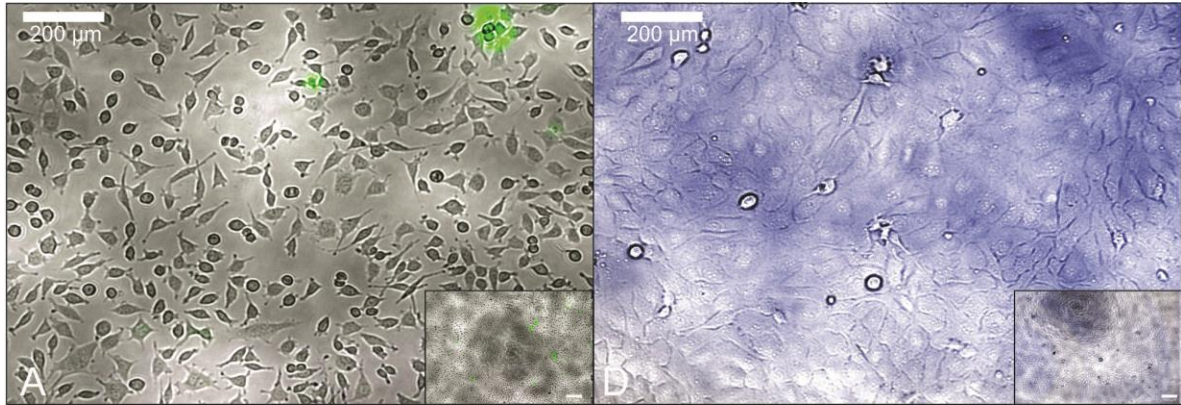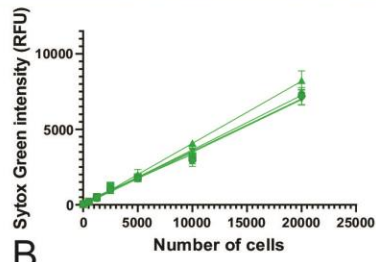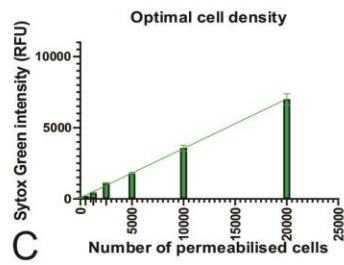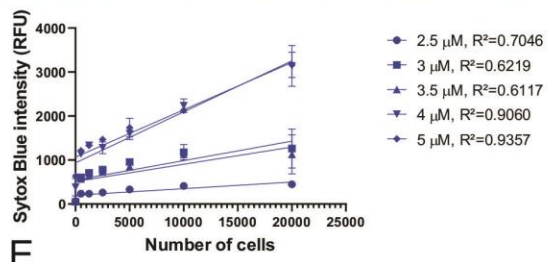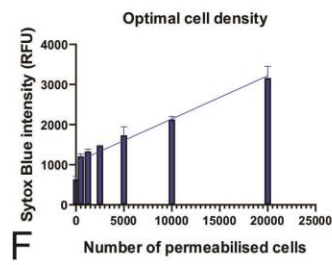

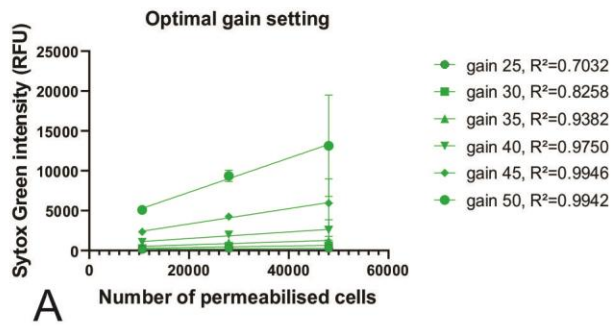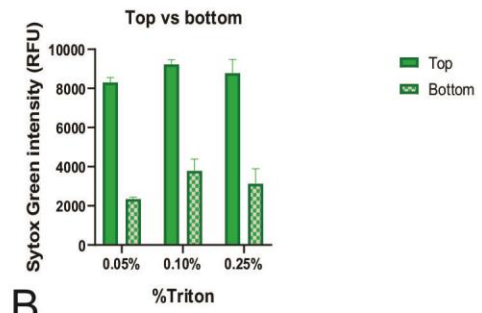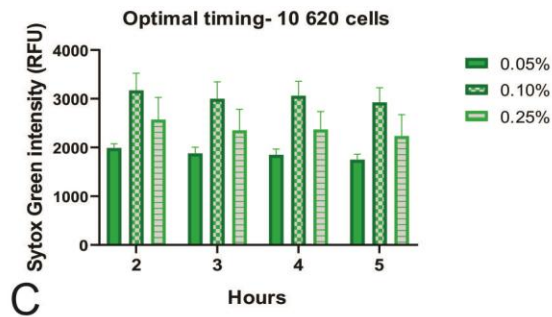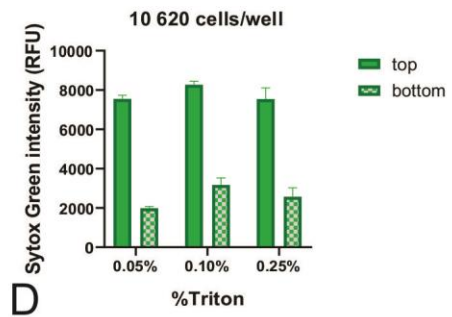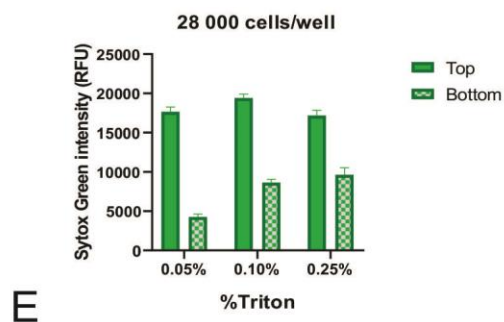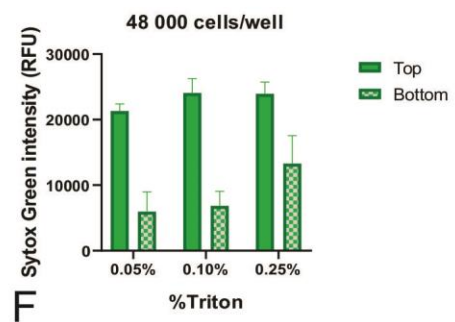

Supplement: Supplementary file 1 [file cells-09-00703-s001.pdf]
